# Biomarkers in Autism

**DOI:** 10.3389/fpsyt.2014.00100

**Published:** 2014-08-12

**Authors:** Andre A. S. Goldani, Susan R. Downs, Felicia Widjaja, Brittany Lawton, Robert L. Hendren

**Affiliations:** ^1^Universidade Federal do Rio Grande do Sul, Porto Alegre, Brazil; ^2^Department of Psychiatry, University of California San Francisco, San Francisco, CA, USA

**Keywords:** biomarker, autism spectrum disorders, epigenetics, treatment targets, neuroimaging, genetics

## Abstract

Autism spectrum disorders (ASDs) are complex, heterogeneous disorders caused by an interaction between genetic vulnerability and environmental factors. In an effort to better target the underlying roots of ASD for diagnosis and treatment, efforts to identify reliable biomarkers in genetics, neuroimaging, gene expression, and measures of the body’s metabolism are growing. For this article, we review the published studies of potential biomarkers in autism and conclude that while there is increasing promise of finding biomarkers that can help us target treatment, there are none with enough evidence to support routine clinical use unless medical illness is suspected. Promising biomarkers include those for mitochondrial function, oxidative stress, and immune function. Genetic clusters are also suggesting the potential for useful biomarkers.

## Introduction

Several neurodevelopmental disorders have complex genetic and epigenetic features that lead to their phenotype and for some there is no single genetic marker for the diagnosis; therefore, the diagnosis is made phenotypically as in schizophrenia, ADHD, and autism spectrum disorder (ASD). While phenotypic characterization of neurodevelopmental disorders is an integral part of advances in clinical practice and research, a given phenotype may arise from a diverse set of biochemical processes (especially when the disorder is caused by numerous genetic and epigenetic factors). Therefore, the treatment of a “phenotypic diagnosis” with a specific drug or intervention might be extremely effective for one “phenotypically characterized” individual with a given set of genetic and/or epigenetic biomarkers, but completely ineffective for another with a different pattern of biomarkers. An important goal of ongoing research in ASD, therefore, is to more precisely identify the many different abnormal genetic and epigenetic processes that underlie the phenotype of the disorder. This might allow individuals with ASD to be characterized into subsets with certain biomarker profiles that would respond more favorably to specific treatments. It also has the potential to elucidate the abnormal physiology that leads to autism, which could improve the understanding of the disorder and lead to earlier diagnosis and more targeted treatments.

A significant challenge in identifying biomarkers in ASD is that biomarkers may reflect genetic and neurobiological changes or epigenetic (broadly defined, see below) processes that may be active only during particular periods of time and do not define the disorder, only the process that led to it. In addition, treatment research should ideally include biomarkers that are believed to predict improvements in clinical symptoms from clinical interventions (1) to know if an intervention is altering or targeting an active biomedical process that relates to response in the subject at that time. Indeed, the National Institute of Mental Health (NIMH) has changed how they fund clinical trials so that “trial proposals will need to identify a target or mediator; a positive result will require not only that an intervention ameliorated a symptom but also that it had a demonstrable effect on a target, such as a neural pathway implicated in the disorder or a key cognitive operation”(2).

Traditionally, research in psychiatry has been guided by DSM symptom based diagnoses and selection criteria for clinical trials were based on these symptom clusters. Biomarkers have not been reliable or valid markers of response to treatment in past trials, and this may be due to the wide variety of genetic and epigenetic processes that underlie the DSM-based diagnosis. Recently, progress in biomarker research has led to the commitment to the Research Domain Criteria project (RDoC) as a basis for future NIMH funding for biomarker based research (3, 4). The RDoC goal is to define basic dimensions of functioning to be studied across multiple units of analysis, from genes to neural circuits to behaviors, cutting across disorders as traditionally defined. The intent is to translate rapid progress in basic neurobiological and behavioral research to an improved integrative understanding of psychopathology and the development of new and/or optimally matched treatments for mental disorders (5).

In this article, we review the literature on biomarkers for ASD including genetic, epigenetic, brain based, and body metabolism biomarkers. This is a huge area and this review is not intended to be comprehensive. New potential biomarkers for ASD are being identified every day so the list needs to be updated frequently. We do extensively review the literature at the time of this writing, report on methodologically sounds studies, offer summary tables, and summarize what we know.

## Genetic Biomarkers

**Figure 1:**
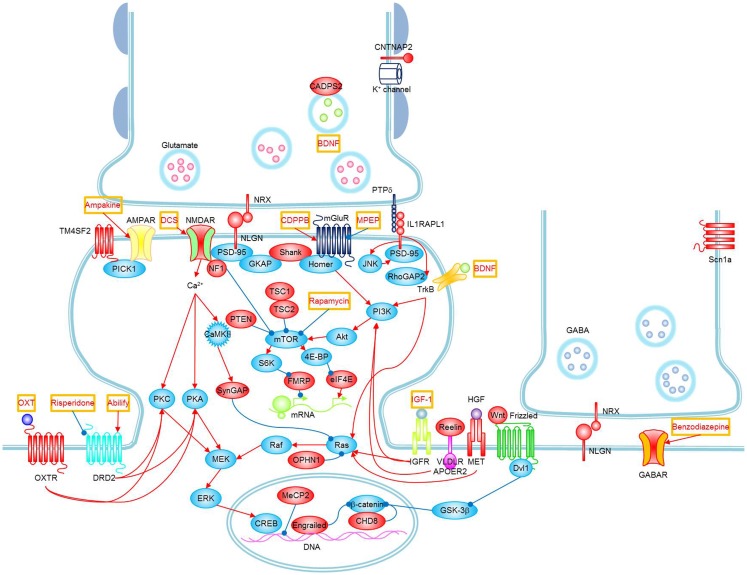
**Signaling pathways and possible treatments associated with ASD**. Molecules whose mutations or polymorphisms are associated with ASD are indicated in red. Stimulations and inhibitions are indicated by red and blue arrows, respectively. Possible treatments and their target molecules are indicated by red texts in orange boxes. SynGAP1, which directly interacts with PSD-95, could not be placed next to PSD-95 for simplicity. Figure as originally published in Won et al. (8).

The literature supports a hereditary component in the susceptibility to ASDs, there are much higher concordance rates of ASDs in monozygotic twins (92%) than dizygotic twins (10%), and a recent estimate of the sibling recurrence risk ratio (λs) is 22 for autism. Despite being highly heritable, ASDs show heterogeneous clinical symptoms and genetic architecture, which have hindered the identification of common genetic susceptibility factors. Although previous linkage studies, candidate gene association studies, and cytogenetic studies have implicated several chromosomal regions for the presence of autism susceptibility loci, they have not consistently identified and replicated common genetic variants that increase the risk of ASDs other than some clearly genetic disorders such as fragile X, tuberous sclerosis, and RASopathies whose phenotypes meet the ASD category description (5). As autism is not a single clinical entity, it can be viewed as a behavioral manifestation of tens or perhaps hundreds of genetic and genomic disorders (6). It has been estimated that there are over 500 distinct genetic loci that may be related to ASD (7) (Figure 1).

In addition, recent research has shown that there are many epigenetic mechanisms that could account for hereditary influences. A study by Hallmayer et al. (9) reports that the environment may actually account for more of the etiology of autism than genetics. Their study, the largest population-based twin study of autism that used contemporary standards for the autism diagnosis, found that heritability estimated at 38%, while shared environmental component was 58% (9). Heritability of ASD and autistic disorder is estimated to be approximately 50% (10).

Being one of the most familial psychiatric disorders, autism has garnered inquiries about possible genetic biomarkers (11); however, progress has been slow until recently with the introduction of genome-wide association studies (GWAS) and microarrays (12). Research into the microbiological underpinnings of ASDs suggests that it is not a monogenic disorder following Mendelian tendencies, with a few studied individuals and families as notable exceptions (11). In fact, the literature suggests that the risk of developing autism is derived by variations across many genes, none of which have been conclusively, definitively responsible for ASDs although some individuals with single gene disorders such as fragile X also meet the criteria for ASD.

Genome-wide association studies have identified, with replication, *de novo* variations that are strongly associated (with sufficient power) with ASDs (Table 1): deletions at the Neurexin 1 (NRXN1) locus, duplications at 7q11.23, duplications at 15q11-13, and deletions and duplications at 16p11.2. Earlier studies found rare, functional mutations in genes encoding for NRXN1, SHANK3, and SHANK2, all of which are proteins that affect the functioning of synapses and have been linked to other, known genetic disorders (12). In addition, whole exome sequencing verified by four reports have found genetic mutations associated with autism including SNC2A, CHD8, DYRKIA, POG2, GRIN2B, and KATNAL2 (13).

**Table 1:** **Genetic biomarkers in ASD (see text for references)**.

Neurexin 1 (NRXN1) deletion
7q11.23 duplication
15q11-13 duplication
16p11.2 duplication and deletion
SHANK 3
SHANK 2
SNC2A
CHD8
DYRKIA
POG2
GRIN2B
KATNAL2
CNTN4 deletion
CNTNAP2
5p14.1
CDH10
CDH9
MTHFR 677 > T
SEMA5A
TAS2R1
2q22.1
3p26.3
4q12
14q23
NLGN4

Studying particular genes in certain, recognized disorders with social deficits, such as fragile X syndrome and tuberous sclerosis, may shed light on the genetic underpinnings of ASDs. This strategy gives credence to the idea that ASD is the result of many variations among genes that converge to a similar phenotype. A prime example of implementation of such a strategy is with contactin 4 (CNTN4), and its association with social and intellectual disability in a recurrent deletion syndrome. Mutations in the respective genes are identified in idiopathic ASDs. Similarly, mutations in CNTNAP2 are linked to a variety of results, such as language delay, functional connectivity abnormalities, selective mutism, and anxiety. More importantly in the scope of ASDs, alterations in CNTNAP2 are noted in consanguineous pedigrees (12). Research shows an increased prevalence of ASDs in families that are consanguineous (11).

In a study published by Nature in 2009, Wang and colleagues completed a genetic analysis in a large number of ASD individuals and families, with a combined sample set of more than 10,000 subjects of European ancestry. They identified common genetic variants on 5p14.1 that are associated with susceptibility to ASDs and replicated these findings in separate analyses. The contribution of chromosome 5p14 to cell adhesion and its connection to autism susceptibility supports the conclusion that specific genes in this class help create the connectivity and structure of the brain that ultimately leads to ASD (14). Besides the potential role of the nearby CDH10 and CDH9 genes, pathway-based association analysis lend further support to neuronal cell-adhesion molecules in conferring susceptibility to ASDs, suggesting that specific genetic variants in this gene class may be involved in shaping the physical structure and functional connectivity of the brain that leads to the clinical manifestations of ASDs (14).

Among the common polymorphisms found to be associated with autism risk, the methylenetetrahydrofolate reductase (MTHFR) polymorphism is one of the most widely studied genetic correlations with autism. The MTHFR 677C > T polymorphism causes a reduction in enzyme activity, which results in higher production of 5-formyltetrahydrofolate (5-FTHF) necessary for DNA synthesis and repair along with lower 5-MTHF production. The MTHFR 677C > T polymorphism causes decline of normal enzyme activity to 35% (15). The MTHFR 677T-variant allele is correlated with a 2.79-fold increased risk for autism. However, this study also found that MTRR 66A and SHMT 1420T alleles demonstrated protective roles against autism risk (16). MTHFR also has a strong interaction with maternal folic acid intake before and during pregnancy, which is associated with autism risk. Children with high autism risk whose mothers carried MTHFR 677 TT allele and were reported taking prenatal vitamins had fewer diagnoses of autism than the children whose mothers with the same allele and did not take prenatal vitamins (17).

In several GWAS (14, 18–20), four genes have been associated with ASDs. These genes, cadherin (CDH9), cadherin 10 (CDH10), semaphorin 5A (SEMA5A), and taste receptor, type 2, member 1 (TAS2R1), are found on chromosome 5p14, which regulates axon growth and cell adhesion. While gene networks could not be established from the small number of genes, these findings do suggest that these genes and the dysregulation of synaptic connection may be a key feature in ASDs (21).

Griswold and coworkers found a significantly higher burden in the number and size of deletions carried by ASD individuals when compared with controls (22). Among the copy-number variations (CNVs) identified were several that overlapped with well-established autism-associated regions and candidate genes. They isolated four large, novel deletions on 2q22.1, 3p26.3, 4q12, and 14q23 that include new genes and regions linked to ASDs. Scattered findings related to NLGN4 and autism susceptibility occur across cultures. In the Chinese ASD cases, there were no significant findings regarding SNPs along NLGN4 gene and autism risk (23), yet in Greek ASD cases, nine nucleotide changes in NLGN4X are found to be associated with autism (24).

Copy-number variations has unveiled the overexpression of rare, *de novo* structural variations in the genome of simplex families (families which have one affected offspring) when compared to families with multiple affected offspring, and especially control families. Furthermore, these results have been replicated in later studies, bolstering the confidence in which discoveries can be made about genetic ties with common diseases and autism (12); however, *de novo* CNVs have been found in only 5–10% of researched subjects, and thus, do not make up the majority of affected, researched individuals. Despite this finding, it seems as though large (>100 kb), multigenic *de novo* CNVs are the most indicative of ASD risk at this time.

The genetic component of a disorder can be transmitted or acquired through *de novo* (“new”) mutations. A study based on a 343 family subset of the Simons Simplex collection did not find significantly greater numbers of *de novo* missense mutations in affected versus unaffected children, but gene-disrupting mutations (nonsense, splice site, and frame shifts) were twice as frequent (59 versus 28) (25). They found that the father is more frequently the parent of origin for *de novo* mutations than the mother (50/17) for single nucleotide variants (SNVs). Parental age also appears to play a role in mutation rate. A study published in *Nature* found that the rate of *de novo* SNVs increases with paternal age (*p* = 0.008) and that paternal and maternal ages are highly correlated (*p* < 0.0001) (26). Overall these data demonstrate that non-synonymous *de novo* SNVs, and particularly highly disruptive nonsense and splice-site *de novo* mutations, are associated with ASD.

Several companies are marketing genetic testing for autism based on clusters of genes with a strong clustering for ASD risk (27, 28). In the future, there may be biomarkers that can pinpoint for high risk for ASD diagnosis. For example, a mother who may be high risk for immune dysfunction leading to ASD in a second child once the first child has ASD (29) or the increase in the Akt-mTOR pathway, which can be seen in fragile X syndrome and in other ASD subtypes (30).

### Epigenetics

Considerable symptom severity differences within ASD-concordant monozygotic twins, strongly implicates a role for non-genetic epigenetic factors (31). Epigenetics refers to the study of heritable changes in gene activity that are not caused by changes in the DNA sequence; it also can be used to describe the study of stable, long-term alterations in the transcriptional potential of a cell that are not necessarily heritable. Epigenetic changes in ASD occur through methylation, histone modification (31), chromatin remodeling, transcriptional feedback loops, and RNA silencing (32). Processes in the gene × environment interaction that influence gene expression include metabolic processes such as oxidative stress, mitochondrial function, methylation, immune function, and inflammation that are byproducts of influences such as the mothers and fathers immune systems, environmental toxicants, and diet to name a few. This section will review these epigenetic influences associated with ASD.

Studies show that DNA methylation differences can occur in many loci including AFF2, AUTS2, GABRB3, NLGN3, NRXN1, SLC6A4, UBE3A (31), the oxytocin receptor (33), MeCP2 (a cause for most cases of Rett syndrome) in the frontal cortex (34), and changed chromatin structure in prefrontal cortex neurons at hundreds of loci (35). The severity of the autistic phenotype is related to DNA methylation at specific sites across the genome (31). Environmental and physiological influences are important factors accounting for interindividual DNA methylation differences, and these influences differ across the genome (36). The following sections describe markers for metabolic pathways and environmental influences that can effect epigenetic changes.

## Metabolic Biomarkers

There are no autism-defining, metabolic biomarkers, but examining the biomarkers of pathways associated with ASD can point to potentially treatable metabolic abnormalities and provide a baseline that can be tracked over time. Each child may have different metabolic pathologies related to SNPs, nutrient deficiencies, and toxic exposures. Examples of metabolic disorders that can lead to an autistic-like presentation include phenylketonuria (PKU) (37), disorders of purine metabolism (38), biotinidase deficiency (39), cerebral folate deficiency (40), creatine deficiency (41), and excess propionic acid (which is produced by *Clostridium*) (42, 43).

A recent review assessed the research on physiological abnormalities associated with ASD (44). The authors identified four main mechanisms that have been increasingly studied during the past decade: immunologic/inflammation, oxidative stress, environmental toxicants, and mitochondrial abnormalities. In addition, there is accumulating research on the lipid, GI systems, microglial activation, and the microbiome, and how these can also contribute to generating biomarkers associated with ASD (45, 46).

Pathways are interconnected with a defect in one likely leading to dysfunction in others. Many metabolic disorders can lead to endpoints such as impaired methylation, sulfuration, and detoxification pathways and nutritional deficiencies. Mitochondrial dysfunction, environmental risk factors, metabolic imbalances, and genetic susceptibility can all lead to oxidative stress (47), which in turn leads to inflammation, damaged cell membranes, autoimmunity (48), impaired methylation (49), cell death (48), and neurological deficits (50). The brain is highly vulnerable to oxidative stress (51), particularly in children (52) during the early part of development (47). As environmental events and metabolic imbalances affect oxidative stress and methylation, they also can affect the expression of genes.

Several studies have detected altered levels of a large collection of substances in body-based fluids from ASD subjects compared to controls (e.g., serum, whole-blood, and CSF) (53). These findings encompass either of two main disease-provoking mechanisms: a CNS disorder that is being detected peripherally [e.g., serotonin and its metabolites, sulfate (54), low platelet levels of gamma-aminobutyric acid (GABA) (55), low oxytocin (which affects social affiliation) (56), and low vitamin D levels (57, 58)] or a systemic abnormality that has repercussions in the brain (59).

Serotonin in the brain promotes prosocial behavior and correct assessment of emotional, social cues (60) and can contribute to immune abnormalities (61). Oxytocin can affect social affiliation and social communication deficits (62). Vitamin D has many effects including regulating serotonin synthesis, reducing maternal antibodies that attack the fetal brain, modulating oxytocin synthesis, lowering GI inflammation by lowering gut serotonin (58), DNA repair, anti-inflammatory actions, anti-autoimmune activities, antiseizure activity, increase in regulatory T cells, mitochondrial protection, stimulation of antioxidant pathway (63), and increasing glutathione (64).

### Oxidative stress markers

Oxidative stress can be detected by studying antioxidant status, antioxidant enzymes, lipid peroxidation, and protein/DNA oxidation, all of which have been found to be elevated in children with autism (Table 2). Different subgroups of children with ASD have different redox abnormalities, which may arise from various sources (65). A recent meta-analysis from 29 studies of blood samples from subjects with ASD shows that reduced levels of glutathione, glutathione peroxidase, methionine, and cysteine along with increased levels of oxidized glutathione are statistically different in ASD (66). The level of antioxidants excreted in urine was found to be significantly lower than normal in autistic children. These findings correlated with the severity of the ASD (67).

**Table 2:** **Oxidative stress biomarkers in ASD (see text for references)**.

Glutathione – reduced/oxidized
Methionine
Cysteine
Organic acid test – alpha hydroxybutyrate, pyroglutamate, and sulfate
Plasma F2t-isoprostanes (F2-IsoPs)
Urine8-OHdG
Transferrin
Ceruloplasmin
Plasma 3-chlortyrosine (3CT)
3-Nitrotyrosine (3NT)

Measurements of antioxidant status include measurement of *glutathione*, the primary antioxidant in the protection against oxidative stress, neuroinflammation, and mitochondrial damage (68, 69). Glutathione is instrumental in regulating detoxification pathways and modulates the production of precursors to advanced glycation end products (AGEs) (70). Measuring reduced glutathione, oxidized glutathione, or the ratio of reduced glutathione to oxidized glutathione helps determine the patient’s oxidation status. In many patients with ASD, the ratio of reduced glutathione to oxidized glutathione is decreased, indicating a poor oxidation status (71).

The enzyme glutathione peroxidase has been used as a marker and is typically reduced. There are mixed results concerning the enzyme levels of *superoxide dismutase (SOD)* (72). Other markers for glutathione inadequacy include alpha hydroxybutyrate, pyroglutamate, and sulfate, which can be assessed in an organic acid test. Lipid peroxidation refers to the oxidative degradation of cell membranes. There is a significant correlation between the severity autism and urinary lipid peroxidation products (67), which are increased in patients with ASD.

*Plasma F2t-Isoprostanes (F2-IsoPs)* are the most sensitive indicator of redox dysfunction and are considered by some to be the gold standard measure of oxidative stress (73). They are increased in patients with ASD and are even higher when accompanied by gastrointestinal dysfunction (73). F2t-isoprostanes (F2-IsoPs) can be measured in the urine as well.

*Urine 8-OHdG* is biomarker for oxidative damage to DNA. It is commonly used although there are confounding factors and intra individual variations (74) and some researchers have reported that the increases in urine 8-OHdG in patients with ASD is not significant. The increases in urine 8-OHdG did not reach statistical significance (75).

Decreased levels of major antioxidant serum proteins *transferrin* (iron-binding protein) and *ceruloplasmin* (copper binding protein) have been observed in patients with ASD. The levels of reduction in these proteins correlate with loss of previously acquired language (47) although there are mixed reviews of the significance of this (66).

Plasma *3-chlortyrosine (3CT)*, a measure of reactive nitrogen species and myeloperoxidase activity, is an established biomarker of chronic inflammatory response. Plasma 3CT levels reportedly increased with age for those with ASD and mitochondrial dysfunction but not for those with ASD without mitochondrial dysfunction (65).

3-Nitrotyrosine (3NT) is a plasma measure of chronic immune activation and is a biomarker of oxidative protein damage and neuron death. This measure correlates with several measures of cognitive function, development, and behavior for subjects with ASD and mitochondrial dysfunction but not for subjects with ASD without a mitochondrial dysfunction (65).

### Mitochondrial dysfunction markers

Mitochondrial dysfunction is marked by impaired energy production. Some children with ASD are reported to have a spectrum of mitochondrial dysfunction of differing severity (44) (Table 3). Mitochondrial dysfunction, most likely an early event in neurodegeneration (76), is one of the more common dysfunctions found in autism (77) and is more common than in typical controls (78). There is no reliable biomarker to identify all cases of mitochondrial dysfunction (79). It is possible that up to 80% of the mitochondrial dysfunction in patients with both ASD and a mitochondrial disorder are acquired rather than inherited (44).

**Table 3:** **Mitochondrial function biomarkers in ASD (see text for references)**.

Lactate
Pyruvate
Lactate/pyruvate ratio
Carnitine (free and total)
Alanine
Quantitative plasma amino acids
Ubiquinone
Ammonia
CD
AST/ALT
CO_2_
Creatine kinase
Aspartate aminotransferase
Serum creatine kinase

Mitochondrial dysfunction can be a downstream consequence of many proposed factors including dysreactive immunity and altered calcium (Ca^2+^) signaling (80), increased nitric oxide and peroxynitrite (68), propionyl CoA (81), malnutrition (82), vitamin B6 or iron deficiencies (83), toxic metals (83), elevated nitric acid (84, 85), oxidative stress (86), exposure to environmental toxicants, such as heavy metals (87–89), chemicals (90), polychlorinated biphenyls (PCBs) (91), pesticides (92, 93), persistent organic pollutants (POPs) (94), and radiofrequency radiation (95). Other sources of mitochondrial distress include medications such as valproic acid (VPA), which inhibits oxidative phosphorylation (96) and neuroleptics (97, 98).

Markers of mitochondrial dysfunction include lactate, pyruvate and lactate-to-pyruvate ratio, carnitine (free and total), quantitative plasma amino acids, ubiquinone, ammonia, CD, AST, ALT, CO_2_ glucose, and creatine kinase (CK) (44). Many studies of ASD report elevations in lactate and pyruvate, others report a decrease in carnitine, while others report abnormal alanine in ASD patients (44) or elevations in aspartate aminotransferase and serum CK (99). Increases in lactate are not specific and may only occur during illness, after exercise or struggling during a blood draw (100).

Rossignol and Frye (44) recommend a mitochondrial function screening algorithm. This includes fasting morning labs of lactate, pyruvate, carnitine (free and total), acyl carnitine panel, quantitative plasma amino acids, ubiquinone, ammonia, CK, AST/ALT, CO_2_, and glucose (44). The interpretation of such a panel and the indications for specific treatments has not yet been established.

### Methylation

The methylation pathway provides methyl groups for many functions, including the methylation of genes, which can result in the epigenetic changes of turning genes on and off (Table 4). This transfer occurs when *S*-adenosylmethionine (SAM) donates a methyl group and is transformed to *S*-adenosylhomocysteine (SAH). SAH can be transferred to homocysteine, which can either be re-methylated to methionine or be transferred by the sulfuration pathway to cysteine to create glutathione. With increased oxidative stress, SAH might be diverted away from the methylation pathway to the sulfuration pathway in order to make more glutathione. This will result in less methionine and less methylation ability.

**Table 4:** **Methylation biomarkers in ASD (see text for references)**.

*S*-adenosylmethionine (SAM)/*S*-adenosylhomocysteine (SAH)
Homocysteine
MTHFR

Impaired methylation may reflect the effects of toxic exposure on sulfur metabolism. Oxidative stress initiated by environmental factors in genetically vulnerable individuals, can lead to impaired methylation and neurological deficits (49) both of which may contribute to the manifestation of autism (71).

A marker of methylation dysfunction is decreased SAM/SAH ratio in patients with ASD. Fasting plasma methionine decreases since through SAM it is the main methyl donor. Fasting plasma cysteine, a sulfur containing amino acid is the rate-limiting step in the production of glutathione and is significantly decreased. Plasma sulfate is decreased, which may impair detoxification pathways. Homocysteine is generally increased, but the studies are mixed (66). Vitamin B12 and folate are required for the methylation pathway. The MTHFR genetic SNP is reported to heavily influence the methylation pathway (66).

### Immune dysregulation

#### Cytokine evaluation

Chronic inflammation and microglia cell activation is present in autopsied brains of people with ASD (101, 102) (Table 5). Factors that increase the risk of activating brain microglia include traumatic brain injury (TBI) (103) reactive oxygen species (104) and a dysfunctional blood brain barrier (105). The blood brain barrier can be compromised by oxidative stress (106), acutely stressful situations (107), elevated homocysteine (108), diabetes (109), and hyperglycemia (110). Cytokines can pass through a permeable blood brain barrier and start this process (111). Hence, cytokines can serve as a marker of the immune dysregulation, which can further complicate ASD.

**Table 5:** **Immune biomarkers in ASD (see text for references)**.

Subjects with ASD
TGF-beta
CCL 2
CCL 5
IGM
IgG
Th1/Th2
Neopterin
S110B protein
Anti ganglioside M1 antibodies
Antineronal antibodies
Serum anti-nuclear antibodies
BDNF
Mothers of subjects with ASD
IFN-Y
Il-4
Il-5
Il-6

Irregular cytokines profiles are found in ASD (112, 113) and elevations in plasma cytokines are reportedly correlated with regressive onset and severity of autistic and behavioral symptoms (113). Altered pro-inflammatory cytokines, complement proteins, chemokines, adhesion molecules, and growth factors are correlated with ASD. More specifically, altered TGF-beta, CCL2, and CCL5, IgM and IgG classes of immunoglobulin circulating levels are linked with a worsening of behavioral scores (114). An imbalance in Th1/Th2 has are found as well, which may play a role in the pathogenesis of autism (115).

*Neopertin* as a urine marker of immune dysfunction and activation. Neopterin is associated with increased production of reactive oxygen systems and can be considered as a measurement of the oxidative stress elicited by the immune system. Neopterin levels are found to be significantly higher in children with autism than in the comparison subjects (116).

Increased *S100B protein*, a calcium binding protein produced primarily by astrocytes, is a biomarker reflecting neurological/brain damage found elevated in ASD and correlated to autistic severity (117).

### Autoimmunity and maternal antibodies

Autoimmune autistic disorder is proposed as a major subset of autism (118), and autoimmunity may play a role in the pathogenesis of language and social developmental abnormalities in a subset of children with these disorders (119). There are many autoantibodies found in the nervous system of children with ASD who have a high level of brain antibodies (120, 121). These can be measured as biomarkers in this subset of ASD patients. The anti ganglioside M1 antibodies (122), antineuronal antibodies (123), and serum anti-nuclear antibodies (123, 124) correlate with the severity of autism. Other autoantibodies postulated to play a pathological role in autism include: anti neuron-axon filament protein (anti-NAFP) and glial fibrillary acidic protein (anti-GFAP) (125), antibodies to brain endothelial cells and nuclei (119), antibodies against myelin basic protein (126, 127), and anti myelin associated glycoprotein, an index for autoimmunity in the brain (128). BDNF antibodies were found higher in ASD (129), and low BDNF levels may be involved in the pathophysiology of ASD (130).

Antibodies in patients with autism are found to cells in the caudate nucleus (131), cerebellum (132, 133), hypothalamus and thalamus (121), the cingulate gyrus (134), and to cerebral folate receptors (135). Children with cerebellar autoantibodies had lower adaptive and cognitive function as well as increased aberrant behaviors compared to children without these antibodies (132).

### Mother’s immune status

Research studies indicate an association between viral or bacterial infections in expectant mothers and their ASD offspring (136, 137). Maternal antibodies cross the underdeveloped blood brain barrier of the fetus (138) leading to impaired fetal neurodevelopment and long-term neurodegeneration, neurobehavioral, and cognitive difficulties (139).

A maternal infection or immune response includes cytokines, which affect aspects of fetal neurogenesis, neuronal migration (140), synaptic plasticity, and stem cell fate (141). Elevated serum IFN-γ, IL-4, and IL-5 were more common in women who gave birth to a child subsequently diagnosed with ASD (142). Fetal IL-6 exposure, especially in late pregnancy, leads abnormalities of hippocampal structural and morphology, and decreased learning during adulthood (139).

Some of the antibodies that cross the fetal developing blood brain barrier recognize and attack the brain (138). The presence of fetal brain protein antibodies in ASD can result in an inappropriate approach to unfamiliar peers (143).

Braunschweig et al. developed a panel of clinically significant maternal autoantibody-related autoantibody biomarkers with over 99% specificity for autism risk (144). This panel is suggested to lead to an early diagnosis of maternal autoantibody-related autism, allow for interventions that limit fetal exposure to these antibodies and allow for early behavioral intervention.

### Dysbiosis

When the gut becomes inflamed, it breaks down and becomes permeable, sometimes referred to as dysbiosis. Dysbiosis is reported to be an upstream contributing factor to autoimmune conditions and inflammation. Markers under consideration include circulating antibodies against tight junction proteins, LPS, actomyosin (145) calprotectin (146), and lactoferrin (147). Dysbiosis was found in 25.6% of patients with ASD (148). It is proposed to have a direct effect on the brain as it is a hypothesized source of inflammation (149–151) and autoimmunity (152, 153), possibly through molecular mimicry (154). Diet is one source of dysbiosis (155).

### Amino acids and neuropeptides

Platelet hyperserotonemia is considered one of the most consistent neuromodulator findings in patients with ASD (Table 6). As for other neuropeptides, a recent review reported approximately 15 components that are altered in ASD compared to controls (53). Among them, interesting research has been done on glutamate, GABA, BDNF, and dopamine and noradrenaline systems. A recent study reported a positive correlation between severity of clinical symptoms and plasma GABA levels in patients with ASD, supporting the idea of a disrupted GABAergic system (156). Additionally, a similar grouping of substances measured in the urine is suggested as a more convenient and less invasive way to draw information on these patients (41).

**Table 6:** **Other potential biomarkers in ASD**.

Glutamate
GABA
BDNF
RBC fatty acids

### Fatty acid analysis

Abnormal fatty acid metabolism may play a role in the pathogenesis of ASD and may suggest some metabolic or dietary abnormalities in the regressive form of autism (42, 157). There is evidence of a relationship between changes in brain lipid profiles and the occurrence of ASD-like behaviors using a rodent model of autism (42). Hyperactivity in patients was inversely related to the fluidity of the erythrocyte membrane and membrane polyunsaturated fatty acid (PUFA) levels (158). Imbalances of membrane fatty acid composition and PUFA loss can affect ion channels and opiate, adrenergic, insulin receptors (159) and the modulation of (Na + K)-ATPase activity (160). Analysis of red blood cell membrane fatty acids is a very sensitive indicator of tissue status and may reflect the brain fatty acid composition (161).

Seventeen percent of children with ASD manifest biomarkers of abnormal mitochondrial fatty acid metabolism, the majority of which are not accounted for by genetic mechanisms (162). Patients with ASD had reduced percentages of highly unsaturated fatty acids (163) and an increase in ω6/ω3 ratio (158).

### Environmental toxicants

For environmental toxicant biomarkers, it is difficult to interpret abnormal levels in ASD. For instance, a high burden of aluminum, cadmium, lead, mercury, and arsenic was found in a subgroup of a sample of over 500 patients with ASD (164). Other studies have described decreased levels of some of these heavy metals in urine and in hair samples, which may imply that the body is not excreting the heavy metals adequately (41).

A systematic review of toxicant-related studies in ASD found that pesticides, phthalates, PCBs, solvents, toxic waste sites, air pollutants, and heavy metals were implicated in ASD, with the strongest evidence found for air pollutants and pesticides (165).

## Brain Focused Biomarkers

### Magnetic resonance imaging

Like other areas in psychiatry, new approaches are being devised to tackle ASD in a “bottom-up paradigm” – that is, identifying genetic or biological alterations, which are associated with the clinical manifestations of symptoms. In neuroimaging, much progress has been made toward understanding the condition, but only very few observed biomarkers have sufficient evidence to suggest that they might hold diagnostic or treatment significance.

One of the best-replicated brain findings from subjects with ASD is an early-accelerated brain volume growth. The increase is usually around 10%, peaking between 2 and 4 years of age followed by a plateau (166). Head circumference (HC), an adequate proxy for brain size, is being investigated for diagnostic relevance for ASD (167). However, recent findings on HC in ASD show that there might be an unrelated growth in HC in both patients and controls. Thus, the abnormal overgrowth observed in older studies might be because of a biased Center for Disease Control (CDC) HC norm, which is commonly used as the control group (168).

Gray matter thickness and surface areas and white matter integrity are also being studied. A general trend demonstrating increased gray matter thickness in subjects with ASD compared to controls is observed with an age-dependent effect (166). Even though there are studies correlating symptom severity with altered thickness there are several limitations such as using a cross-sectional approach and a small number of subjects that hinder clinical application (169). Likewise, diffusion tensor imaging (DTI) studies on white matter connectivity are not yet conclusive across studies.

Early studies using functional magnetic resonance imaging (fMRI) focus on task specific cognitive networks (e.g., face recognition, theory of mind, imitation, language processing, and proxies for receptive behavior) (166). In these cognitive network studies, individuals with ASD and controls perform a task while the fMRI is monitored. More recently, researchers are investigating the connectivity between these network and resting-state methods where fMRI is obtained while a subject is at rest and not performing a task. These more recent studies reveal a pattern that suggests less activity in the brain areas that typically perform executive function tasks (such as organization or planning). This combination of activity patterns in ASD is often called a “high noise-information ratio,” supporting an excitatory/inhibitory imbalance theory of ASD (170). Conversely, even though all these fMRI findings shed light on the pathophysiology of ASD, they also are not mature enough to translate into a reliable biomarker that can be used in clinical practice.

### Electroencephalography

Aligned with the notion that ASD is an abnormal connectivity disorder, studies using electroencephalography (EEG) have explored local changes in signal complexities in patients (171). Some studies were able to detect abnormalities as early as 6 months of age, suggesting an important tool for early detection and risk group assessment (172). However, despite findings like multi-scale entropy differences being proposed as an early diagnostic biomarker, EEG has not yet been established as a reliable tool for diagnosis or to document clinical changes (173).

### Neurochemistry

Neuroimaging techniques also are used to monitor *in vivo* concentration of substances in the brain, and include positron emission tomography (PET), single photon emission tomography (SPECT), and magnetic resonance spectroscopy (MRS). So far, the majority of studies report abnormalities in several of neurotransmitter networks and their respective metabolites (e.g., dopamine, GABA, serotonin, glutamate, and *N*-acetyl-aspartate), varying from synthesis, transport, and receptor activity in different regions of the brain in the glutamate–glutamine system, in particular, there appears to be either hyper (174) or hypoglutamatergic (175) states depending on the brain region, which could be interpreted as an excitatory increase relative to inhibition in key neural circuits (176). In addition, studies pointing toward GABA alterations also are accumulating, with findings of reduced levels of GABA in the frontal lobes of subjects with ASD. Using MRS (177), corroborated the histopathologic research on altered density and distribution of the GABA receptors (178).

## Biomedical Interventions

There are no published studies of interventions for ASD that use neuroimaging or genetic biomarkers in a prospective manner to guide treatment. Biomedical interventions based on body fluid/product biomarkers have been used in a small but growing numbers of well designed, published studies. Several recent reviews summarize these (179–181).

## Future Research Directions

A common feature of all prior studies of these putative biomarkers is that most consist of small samples of patients, and therefore, do not grasp the heterogeneity that characterizes ASD. Also, since they mainly compare subjects with ASD to typically developing controls, it is uncertain whether these biomarker profiles are unique to ASD – they may be present in other neurodevelopmental disorders. A promising new method that is designed to increase specificity of biomarkers in ASD is the multiplex immunoassay, a method that analyzes sets of biomarkers to create a diagnostic profile (182, 183). Furthermore, advances in chromatographic and proteomic techniques are also contributing to the progress of the field, allowing easier assessment of several substances (184, 185).

Thus far, numerous studies examining a diverse set of potential biomarkers have found a large number of genetic, imaging, and metabolic tests that are abnormal in children with ASD compared to control subjects. For most of these measures, it is not yet clear if the abnormal biomarker is a contributing factor to the development of ASD or a result of another underlying abnormality (i.e., causal or merely associated). Not surprisingly, the conclusion is that more studies are needed to further explore these possible mechanisms individually. However, the future in the ASD research might involve a broader view of these biomarkers, which might hold more value in combination than in isolation. As a result of new technological advances, it is possible to use a machine learning technique that is trained to identify complex patterns of data that can be applied to new individuals to make predictions (186). A recent study pooled regional white and gray matter volumes of whole-brain MRI scans in ASD subjects using this computer algorithm program, known as super vector machine. As a result, they could classify a new patient as having an ASD diagnosis or not with a high true positive rate (187). Although exemplified with neuroimaging, this approach could be generalized to other biomarkers (53, 188). In other words, individually insignificant biomarkers when analyzed together might generate a pattern of clinical relevance like diagnosis, severity staging, or response to treatment. These techniques might also be able to identify the most relevant or most predictive biomarkers among the many candidate biomarkers described above.

Although the maxim that “further studies are needed” still holds, ASDs may be witnessing the emergence of clinically relevant biomarkers in the near future.

## Conflict of Interest Statement

The authors declare that the research was conducted in the absence of any commercial or financial relationships that could be construed as a potential conflict of interest.
